# Institutional Experience of Using Andexanet Alfa

**DOI:** 10.7759/cureus.9173

**Published:** 2020-07-13

**Authors:** Sushmita Khadka, Vineela Kasireddy, Pravash Dhakal

**Affiliations:** 1 Internal Medicine, Guthrie Medical Group/Robert Packer Hospital, Sayre, USA; 2 Hematology and Oncology, Guthrie Medical Group/Robert Packer Hospital, Sayre, USA

**Keywords:** andexanet alfa, direct acting anticoagulants

## Abstract

Given their ease of use, safety, and efficacy, direct-acting oral anticoagulants (DOACs) are nowadays widely used in patients with atrial fibrillation or venous thromboembolism, with or without an association with malignancy. Andexanet alfa (andexanet) is a recombinant modified human factor Xa decoy protein that reverses the inhibition of factor Xa. After Food and Drug Administration (FDA) approval in May 2018, andexanet has been used for life-threatening bleeding in patients treated with apixaban or rivaroxaban.

In this article, we present a single institutional retrospective review of patients receiving andexanet alfa at Guthrie Robert Packer Hospital. A total of four patients in a period of 10 months received andexanet for intracranial bleeding, 50% (2) had excellent hemostasis, 30 days mortality was 75% (3), and 25% (1) had a thromboembolic event. Anticoagulation was never started in all patients. This review tends to show the real-world utilization data of andexanet in a community hospital setting.

## Introduction and background

Anticoagulant drugs are widely used in the management of patients with venous thromboembolism, atrial fibrillation, and mechanical heart valves. The newer direct-acting oral anticoagulants (DOACs) are now being widely being used given their ease of use (not requiring monitoring of coagulation parameters), safety, and efficacy. When compared to warfarin, apixaban is found to be superior while rivaroxaban and edoxaban are found to be non-inferior in clinical trials, including in patients with atrial fibrillation [1-3]. Similarly, edoxaban, rivaroxaban, and apixaban were shown to be non-inferior as compared to dalteparin in malignancy-associated venous thromboembolism (VTE) [4-6]. Estimated major bleeding episodes with rivaroxaban ranged from 2.2-4.3 per 100 patients a year [7-8]. With the increasing use of newer anticoagulants, the incidence of bleeding complications is expected to rise. In 2015, idarucizumab was approved for dabigatran (direct thrombin inhibitor) but there was an unmet need for a reversal agent for factor Xa inhibitor. Initially, a four-factor prothrombin complex concentrate was proposed, but it was not proved to be effective [9].

Andexanet alfa (andexanet) is a recombinant modified human factor Xa decoy protein that has been shown to reverse the inhibition of factor Xa in healthy volunteers. It binds and sequesters factor Xa inhibitors in the active site with high affinity, thereby restoring the activity of endogenous factor Xa and reducing levels of anticoagulant activity, as assessed by the measurement of thrombin generation and anti-factor Xa activity, the latter of which is a direct measure of anticoagulant activity [10-11]. The side-effect evident in a phase-one study was a mild transfusion reaction, without thrombotic events, whereas in the andexanet alfa in Patients Receiving a Factor Xa Inhibitor Who Have Acute Major Bleeding (ANNEXA-4) study, thromboembolic events were up to 18% [12]. In a phase-2 clinical study, it reduced anti-factor Xa activity by 52% and 72% with 600 mg and 800 mg bolus, respectively, with a duration of effect of an hour, which is the half-life of andexanet [13]. It reduced the level of anti-factor Xa activity by 94% and 96% in healthy volunteers taking apixaban and rivaroxaban, respectively [14]. In May 2018, andexanet was approved by the FDA for use in life-threatening bleeding in patients treated with apixaban or rivaroxaban [15]. It is used when the last dose of factor Xa inhibitor is <8 hours/unknown (if apixaban <5 mg/rivaroxaban <10 mg) or >8 hours irrespective of the dose [16]. There is a black-box warning for arterial and venous thromboembolic events, ischemic events, cardiac arrests, and sudden deaths [17].

In a multicenter, prospective, open-label, single-group study of 352 patients on apixaban or rivaroxaban with life-threatening bleeding (intracranial hemorrhage in 64% and gastrointestinal (GI) bleeding in 26%) were given a bolus of andexanet followed by a two-hour infusion. At 12 hours, 82% of the patients had good hemostasis; in a month, thromboembolism happened in 10% and 14% of the patient died [18].

Since andexanet alfa is relatively new and expensive (average cost of $24,000 to $49,000 for a single dose), it is uncommonly used and needs more real-time data [19]. A single institutional experience of the use of andexanet alfa in 13 patients demonstrated effective hemostasis in 77% of the patients with a 31% incidence of thromboembolic events and a 15% mortality rate. There are few other single institutional case series on its use in a limited number of patients from large tertiary hospitals [20-21]. We hereby report the utilization of andexanet alfa in a rural community hospital through a retrospective review of patients at Guthrie Robert Packer Hospital who received andexanet from June 2019 to March 2020 for the reversal of life-threatening bleeding associated with rivaroxaban or apixaban.

## Review

Case 1

**Figure 1:**
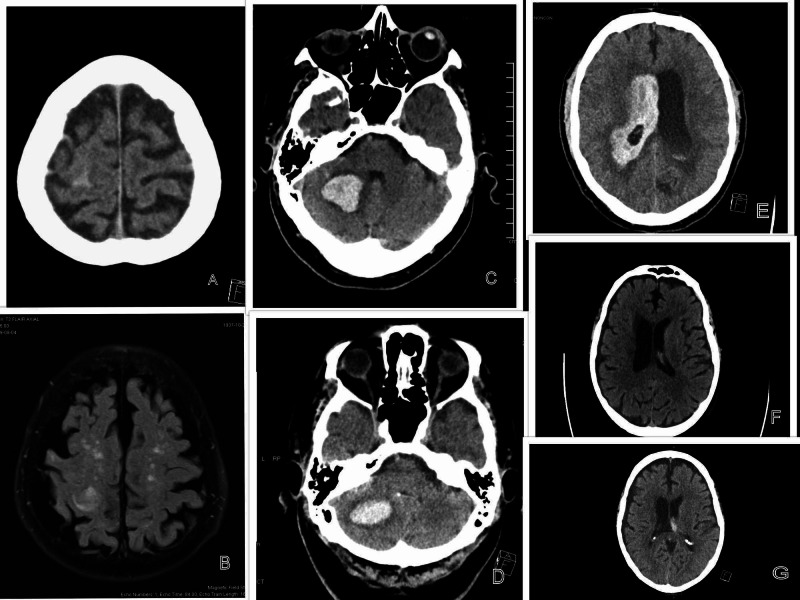
Image showing intracranial hemorrhage in patients at presentation and 12 hours after receiving andexanet (Case 1) A: CT scan at presentation with a small subarachnoid hemorrhage in the right frontoparietal lobe, B: MRI 12 hours later, showing the unchanged subarachnoid hemorrhage in the right frontoparietal region. (Case 2) C: CT scan showing a cerebellar hemorrhage at presentation, D: CT scan at 12 hours with the cerebellar hemorrhage reduced in size. (Case 3) E: CT scan of the head at presentation showing a diffuse intraventricular hemorrhage. (Case 4) F: CT Scan at presentation showing a small amount of hemorrhage in the left intraventricular foramen, G: CT scan at 12 hours showing increased intraventricular hemorrhage in the left intraventricular foramen. CT: computed tomography; MRI: magnetic resonance imaging

An 81-year-old male with a medical history of colon cancer status post colon resection, resident of a long-term care facility, on rivaroxaban 20 mg daily for atrial fibrillation and recent deep vein thrombosis (DVT) presented with left-sided weakness of the extremities and an episode of seizure. She was found to have a small subarachnoid hemorrhage on the right frontal region in the area of the vertex. The patient's rivaroxaban was reversed with a low-dose regimen of andexanet alfa. The patient's mentation and neurological status continued to worsen. Magnetic resonance imaging (MRI) of the brain 12 hours after andexanet continued to show a small subarachnoid hemorrhage (SAH) in the right frontoparietal region, which was unchanged (Figures 1A-1B). Later, a computed tomography (CT) scan of the head at 48 hours showed a significant increase in the hemorrhage with evidence of a midline shift. Given her comorbidities and poor prognosis, no neurosurgical intervention was done, the family decided for comfort care measures, and the patient passed away later.

Case 2

A 71-year-old male, with chronic DVT secondary to factor V mutation and atrial fibrillation, on warfarin, which was changed to rivaroxaban eight months ago after having an ischemic cerebrovascular accident (CVA) presented with ataxia and vertigo and was found to have a cerebellar hemorrhage. The patient initially got Pasteur Culture Collection patient-centered care (PCC) followed by a low dose of andexanet for rivaroxaban reversal. A CT scan of the head 12 hours later shows the stable size of the hemorrhage (Figures 1C-1D). The patient also has acute-on-chronic deep vein thrombosis on the external iliac and femoral veins of the leg, There was also evidence of a pulmonary embolism (PE). An inferior vena cava (IVC) filter was placed. The patient's mental status worsened, the Glasgow Coma Scale (GCS) dropped to <7, there was an aspiration event, and the patient was intubated. The patient ultimately developed refractory shock with multiorgan failure with disseminated intravascular coagulation (DIC). The patient was further transfused with cryoprecipitate and platelets. Since the patient continued to worsen, the family decided on comfort care and the patient died on the seventh day.

Case 3

A 66-year-old male, with significant coronary artery disease (CAD) and atrial fibrillation, on apixaban for anticoagulation, presented with acute onset of headache, vomiting, and altered mental status, prompting intubation on the way to the hospital. A CT scan of the head showed a diffuse intraventricular hemorrhage with communicating hydrocephalus (Figure 1E). CT angiography suspected a ruptured aneurysm in the posterior horn of the right lateral ventricle. The patient received a low dose andexanet regimen and a ventricular drain was placed by neurosurgery. The patient continued to be unresponsive. No repeat imaging of the head was available, as the patient expired on comfort care measures on the third day.

Case 4

A 73-year-old male, with a history of ischemic CVA, residual weakness in the left upper and lower extremities, and atrial fibrillation on apixaban, presented after an episode of a mechanical fall where he hit his head, which was associated with loss of consciousness. The patient was found to alert and oriented at the time of presentation, but the CT scan of the head did show a small hemorrhage in the left interventricular foramen with a chronic right subdural hemorrhage. As the last dose of apixaban was <8 hours prior, the patient received a high-dose regimen of andexanet. CT head 12 hours later showed a worsened intraventricular hemorrhage, but it remained stable in another CT scan in 24 hours (Figures 1F-1G). The chronic subdural hemorrhage remained unchanged, but the patient started improving clinically. His neurological status was closely monitored for nine days in the hospital, and he was discharged with anticoagulation on hold. At the two weeks follow-up, his intraventricular hemorrhage had resolved.

Results

The mean age of the patients was 72 years. All patients (100%) received andexanet for intracranial hemorrhage. Two patients received it for reversal of rivaroxaban and two patients received it for reversal of apixaban (Table 1). Three patients had repeat imaging of the brain in 24 hours, and two had excellent hemostasis (defined as less than 20% increase in the volume of intracranial hemorrhage in one hour and 12 hours after the infusion of andexanet) [18]. Three patients received a low-dose regimen, whereas one patient received a high-dose regimen [17]. The dose of apixaban received was based on the last dose of anticoagulant. One patient who received a low dose of andexanet had new onset of DVT and PE. Three patients went on comfort care, as their clinical status failed to improve, and died eventually. One patient recovered from the event and anticoagulant was never restarted because of the co-existing chronic subdural hematoma (Table 2).

**Table 1:** Baseline characteristics of patients Low-dose regimen: 400 mg IV bolus followed by IV infusion at 4 mg/min for two hours. High-dose regimen: 800 mg IV bolus followed by IV infusion at 8 mg/min for two hours DOAC: direct-acting oral anticoagulant; IV: intravenous

Characteristics	Total patients N= 4
Mean age of the patients	72 years
Male	4
Atrial fibrillation as the primary indication of anticoagulation	4
Comorbidities/past medical history	
Chronic kidney disease	1
Coronary artery disease	3
Cerebrovascular accident	3
Deep venous thrombosis	3
Pulmonary embolism	1
Congestive heart failure	2
Diabetes mellitus	2
DOAC used	
Apixaban	2
Rivaroxaban	2
Last dose of DOAC	
<8 hours	1
>8 hours	3
Intracranial hemorrhage as an indication for the use of andexanet	4
Dose of andexanet alfa used	
Low-dose regimen	3
High-dose regimen	1

**Table 2:** Outcome of the use of andexanet

Outcome measures	Number of patients
Excellent hemostasis	2
30-day mortality	3
Evidence of thromboembolism	1
Restarting of any anticoagulant	0

## Conclusions

Andexanet alfa was used as an effective reversal agent for achieving hemostasis in the majority of patients with a major bleeding episode, and results from our case series are consistent with other existing studies. One patient had a thromboembolic event, likely related to andexanet alfa, but no new adverse events were noted. Despite the rapid reversal of factor Xa, the limiting factors for the use of andexanet alfa is that it is relatively recent on the market and its high cost for a single dose. More real-world studies on its use, including its cost-effectiveness, will greatly add to our knowledge and comfort with using this very important drug.
